# Exploration of contextual factors in a successful quality improvement collaborative in English ambulance services: cross‐sectional survey

**DOI:** 10.1111/jep.12438

**Published:** 2015-08-24

**Authors:** Viet‐Hai Phung, Nadya Essam, Zahid Asghar, Anne Spaight, Aloysius N. Siriwardena

**Affiliations:** ^1^ Community and Health Research UnitSchool of Health and Social CareUniversity of LincolnLincolnUK; ^2^ Clinical Governance Audit, and ResearchEast Midlands Ambulance Service NHS TrustLincolnUK

**Keywords:** health care, leadership, management, organizational theory, pre‐hospital care, quality improvement

## Abstract

**Rationale, aims and objectives:**

Clinical leadership and organizational culture are important contextual factors for quality improvement (QI) but the relationship between these and with organizational change is complex and poorly understood. We aimed to explore the relationship between clinical leadership, culture of innovation and clinical engagement in QI within a national ambulance QI Collaborative (QIC).

**Methods:**

We used a self‐administered online questionnaire survey sent to front‐line clinicians in all 12 English ambulance services. We conducted a cross‐sectional analysis of quantitative data and qualitative analysis of free‐text responses.

**Results:**

There were 2743 (12% of 22 117) responses from 11 of the 12 participating ambulance services. In the 3% of responders that were directly involved with the QIC, leadership behaviour was significantly higher than for those not directly involved. QIC involvement made no significant difference to responders' perceptions of the culture of innovation in their organization, which was generally considered poor. Although uptake of QI methods was low overall, QIC members were significantly more likely to use QI methods, which were also significantly associated with leadership behaviour.

**Conclusions:**

Despite a limited organizational culture of innovation, clinical leadership and use of QI methods in ambulance services generally, the QIC achieved its aims to significantly improve pre‐hospital care for acute myocardial infarction and stroke. We postulate that this was mediated through an improvement subculture, linked to the QIC, which facilitated large‐scale improvement by stimulating leadership and QI methods. Further research is needed to understand success factors for QI in complex health care environments.

## Introduction

Ambulance services are an important component of care pathways for emergencies [1]. In 2005, the UK Department of Health (DoH) report, ‘Taking Healthcare to the Patient’, articulated a national strategic vision for transforming ambulance services to provide a wider range of high‐quality services. This was to be achieved through leadership and cultural change, education, patient and public involvement, and partnerships with other health and social care organizations. A year later, 31 ambulance services merged to form 12 larger regional organizations, at least partly to encourage leadership and cultural change to stimulate innovation and quality improvement [2].

Quality improvement (QI) describes a set of concepts, methods and skills that were initially developed in industry and have subsequently been applied to health care settings. These include the model for improvement, Plan‐Do‐Study‐Act (PDSA) cycles, process mapping and statistical process control [3]. These methods, though successfully applied, have been slow to diffuse into health care and the UK National Health Service (NHS) [4]. Where they have been used, they have had variable effects because of the different contexts in which they have been applied.

Ambulance service chief executive officers promoted national structures for improving clinical quality in 2006 [5]. The National Ambulance Services Clinical Quality Group developed clinical indicators to supplement ‘response time targets’ for quality assurance and improvement in ambulance services. Clinical performance indicators were piloted in 2007 and introduced the following year with the explicit aim of improving quality rather than simply benchmarking: 20 indicators for five clinical conditions included aspirin for suspected acute myocardial infarction (AMI) and face‐arm‐speech test for stroke [6]. Improvement was seen as involving ‘leadership and a move to a quality culture involving front‐line staff in improving care based on implementing evidence’ [6].

Terms such as clinical leadership, organizational culture, innovation and quality are broad in scope, difficult to define and heavily contested notions, but nevertheless important as part of the complex and ‘messy’ context for quality improvement in health services [7].

Clinical leadership is emphasized for improving clinical quality because health care workers are more likely to be influenced by opinion leaders within their own professional group [8, 9], and clinicians have the power to enable or subvert change in practice [10]. Clinical leadership in the NHS, according to the NHS Leadership Academy (Box 1), exists when ‘… clinicians can contribute to the leadership task where and when their expertise and qualities are relevant and appropriate to the context in which they work’ [11]. In order to improve quality and safety, it is therefore vital that clinicians are competent leaders [12].

Box 1 Five domains of clinical leadership*

**Table:** 

Demonstrating personal qualities Developing self‐awareness Managing yourself Continuing personal development Acting with integrity Working with others Developing networks Building and maintaining relationships Encouraging contribution Working within teams Managing services Planning Managing resources Managing people Managing performance Improving services Ensuring patient safety Critically evaluating Encouraging improvement and innovation Facilitating transformation Setting direction Identifying the contexts for change Applying knowledge and evidence Making decisions Evaluating impact

* From the NHS Leadership Academy (2011) Clinical Leadership Competency Framework

Organizational culture [13] has been conceived as a set of ‘shared beliefs, attitudes, values, and norms of behaviour’ of staff, and the structures or processes (so‐called ‘artefacts’), which are manifestations of these, and are amenable to change [14]. Innovation is often a prerequisite for improvement and has been defined as ‘… the intentional introduction of processes and procedures, new to the unit of adoption (team or organisation) and designed to significantly benefit the unit of adoption, staff, patients or the wider public’ [15].

Clinical leadership and organizational culture for improvement also underpinned early notions of clinical governance which has been defined as ‘… a system through which NHS organisations are accountable for continuously improving the quality of their services and safeguarding high standards of care by creating an environment in which excellence in clinical care will flourish’ [16]. Both leadership and organizational culture are important contextual factors for clinical governance. They are necessary, but not sufficient in themselves, for the success or failure of QI initiatives [7, 17]. Whichever way they are operationalized, the relationship between clinical leadership, organizational culture, successful QI and organizational performance is complex and poorly understood [18].

Quality improvement collaboratives (QICs) provide a unique opportunity to study clinical leadership, organizational culture and performance since they involve all three. QICs are a way of ‘testing and implementing evidence‐based changes quickly across organisations' [19]. Their explicit aim in health care is to improve quality in a specific area of practice, with expert support, involving multi‐professional teams from multiple sites working collaboratively and using QI methods [20].

The Ambulance Services Cardiovascular Quality Initiative (ASCQI) was a national QIC involving all 12 ambulance services in England between January 2010 and February 2012 [21]. It aimed to improve care bundles for AMI from 43% to at least 70% and for stroke from 83% to at least 90% through engagement in the collaborative and sharing learning through professional networks within and across services. The results previously published showed that overall performance for the AMI care bundle increased to 79% and for stroke to 96% [21].

In this study, we aimed to explore the relationship between clinical leadership behaviour, organizational culture of innovation and clinical engagement in QI among ambulance clinicians participating in this large‐scale national ambulance QIC.

## Methods

### Study design

We used a self‐administered online questionnaire survey to gather quantitative data for a cross‐sectional analysis and also undertook qualitative analysis of free‐text responses. The design enabled simultaneous collection of quantitative and qualitative data, since the questionnaire contained predominantly closed but with one open‐ended question. We used mixed methods insofar as the qualitative data were integrated with the quantitative findings to help understand the latter [22].

We sent the questionnaire to paramedics in the 12 ambulance services in England. Eleven of the 12 ambulance services participated in the survey, with one service withdrawing because of operational pressures.

The QIC was based on a programme theory that collaborative teams in each service would undergo education in QI methods (PDSA cycles, process maps, driver diagrams, overcoming barriers to improvement). They would apply these to improve AMI and stroke care bundles in one locality and share learning with colleagues in their service and other services through a series of workshops and online meetings to spread improvement more widely.

Our previously published study showed that only one of the 11 participating ambulance services did not experience any increase in delivering at least one of the two care bundles. Seven of the participating trusts experienced an increase in the delivery of both care bundles, with the remaining four experiencing an increase in the delivery of at least one of the two. These findings illustrated the significant impact of this particular QI intervention and the QIC in delivering improved care [20].

A key secondary objective of the QIC was to spread QI methods throughout English ambulance services and it was in this context that we undertook this study.

### Questionnaire development

We modified an online questionnaire, adapted from a previous study [4], after piloting with a small group of ambulance practitioners. The final survey (Appendix S1) included questions in four domains: demographics (Table 1); leadership behaviour; organizational culture of innovation; and use and effectiveness of QI methods.

**Table 1:** Demographic characteristics and mean scores for reported leadership behaviour, organizational culture of innovation and uptake of QI methods in responders

Ambulance service (*n* = 2743)	*n* (%)	Leadership (% score)	Innovation culture (% score)	Use of QI methods
East Midlands	149 (5.4)	53.8	41.2	10.6
East of England	368 (13.4)	52.6	43.1	9.6
Great Western	156 (5.7)	53.7	40.1	9.3
Isle of Wight	20 (0.7)	48.5	47.6	18.0
London	282 (10.3)	51.4	47.6	6.9
North East	147 (5.4)	51.6	40.3	9.2
North West	370 (13.4)	52.1	44.0	10.0
South Central	227 (8.3)	51.2	47.9	7.8
South West	369 (13.5)	53.3	52.5	9.2
West Midlands	248 (9.0)	53.6	44.9	9.9
Yorkshire	407 (14.8)	50.1	38.8	6.8
Total	2743 (100.0)			
ASCQI membership (*n* = 2741)				
Member	86 (3.1)	56.8	48.6	15.9
Non‐member	2655 (96.9)	51.7	44.5	8.6
Total	2741 (100.0)			
Length of service (years) (*n* = 2743)				
0–4	619 (22.6)	53.0	44.7	7.3
5–9	731 (26.6)	52.1	44.1	7.7
10–14	600 (21.9)	51.8	42.3	10.0
15–19	261 (9.5)	53.6	46.9	11.0
20–24	189 (6.9)	49.4	48.4	9.3
25 +	343 (12.5)	49.4	46.4	8.7
Total	2743 (100.0)			
Job role (*n* = 2741)				
Emergency care assistant (EMT1)	198 (7.2)	53.2	48.2	6.4
Qualified technician (EMT2)	139 (5.1)	44.7	44.4	5.3
Student paramedic	263 (9.6)	52.5	44.0	7.3
Paramedic	222 (8.1)	50.5	41.6	7.6
Emergency care practitioner	1386 (50.6)	55.4	48.8	11.0
Clinical/paramedic team leader	124 (4.5)	56.8	48.7	13.4
Operational manager	306 (11.2)	65.3	57.6	25.6
Other	103 (3.8)	56.5	51.2	12.4
Total	2741 (100.0)			
Number of colleagues worked with (*n* = 2743)				
0–3	221 (8.1)	48.6	42.2	8.9
4–6	663 (24.2)	50.8	44.6	7.9
7–9	500 (18.2)	50.6	46.0	8.7
10 +	1359 (49.5)	53.6	44.5	9.4
Total	2743 (100.0)			

Totals that fall short of 2743 represent missing data.

We measured leadership behaviour by self‐reporting behaviours against 11 items adapted from a longer instrument focusing on two domains, ‘inspiring shared vision’ and ‘challenging the process’. These are considered critical for leading improvement [23]. We then rated leadership behaviour on a five‐point Likert scale (‘never’ to ‘very frequently’; Box 2) [24].

Box 2 **Eleven dimensions measuring leadership behaviour**

How often do you talk to others about future trends that will influence how your work gets done?
How often do you seek out opportunities that test your skills and abilities?
How often do you describe an image to others of what your future ambulance service could be like?
How often do you challenge others to try out new and innovative ways to do their work?
How often do you ask others to share their aspirations of their future within the ambulance service?
How often do you search outside of your organization for ways to improve what you do? (e.g. look at other organizations to see how they work)
How often do you show others how long‐term interests at work can be realized by sharing a common vision (e.g. do you have a clear vision of the future and do you communicate this to others?)
How often do you ask ‘what can I learn?’ when things don't go as expected?
How often do you share with others what you want to achieve in your role?
How often do you make certain that you set achievable goals, make accurate plans and establish measurable milestones, for the tasks you work on?
How often do you speak with your colleagues about the meaning and purpose of your work?

Organizational culture for innovation was measured on seven dimensions: risk, resources; sharing of knowledge; targets, tools and techniques; and rewards and relationships. We used an 11‐point rating scale, ranging from 0 ‘very unsupportive’ through to 10 ‘very supportive’ (Box 3) [25].

Box 3 **Seven dimensions measuring organizational culture of innovation**

**Risk taking**

To what degree does your organization provide support for you to try out something new (given that reasonable precautions to avoid harm to patients or disruptions to the organization have been made)?

**Resources for innovation**

To what degree does your organization provide money, protected time, information and/or authority to act for those who wish, to try new ways of working?

**Widely shared knowledge**

To what degree is knowledge gathered and easily shared throughout your organization?

**Specific targets**

To what degree do your managers make it clear that new and better ways of working are important in areas that are strategically or operationally important to the organization?

**Tools and techniques**

To what degree does your organization actively support and promote the use of quality improvement methods?

**Reward systems**

To what degree does your organization reward the innovative efforts of individuals by giving these people things that they really want? (e.g. more protected time for research, more authority and recognition among peers etc.)

**Rapidly formed relationships**

To what degree does your organization easily form high‐performing teams and networks of motivated individuals?

Responders were asked to rate their own use of QI tools and techniques currently in health service use. We used a four‐point Likert scale with 0 representing ‘never’ and 3 representing ‘very frequently’, to determine the extent of adoption and effectiveness of these methods in ambulance services (Box 4). The questionnaire also contained a free‐text box, inviting responders to give more in‐depth responses on their views on ‘how to achieve and maintain clinical engagement in quality improvement initiatives’.

Box 4 **Quality improvement methods, tools and techniques**

1. Clinical audit
2. PDSA cycles
3. Significant event analysis
4. Root cause analysis
5. SWOT/SCOT analysis
6. Force field analysis
7. Process mapping
8. Process redesign
9. WIFM charts
10. Financial rewards for staff
11. Role redesign
12. Confidence charts
13. Run/control charts
14. Pareto charts
15. Cause and effect diagrams
16. Swim lane diagrams
17. CTQ trees
18. Patient interviews
19. Focus groups
20. Balanced scorecards
21. Lean
22. Six sigma

### Questionnaire distribution

We sent information explaining the purpose and voluntary nature of the survey by email via clinical leads in each trust to all front‐line emergency staff in all 12 ambulance services in England between January and February 2012. The email included a web‐link through Survey Monkey, which directly transferred them to the questionnaire. Three reminders (containing the web‐link address) were sent out fortnightly to maximize the response rate.

### Data analysis

Descriptive statistics illustrated the frequency of responses. We used Cronbach's alpha to assess the internal consistency of the four scales covering four domains derived from the questionnaire: ‘leadership behaviour’; ‘organisational culture of innovation’; ‘use of QI methods’; and (perceived) ‘effectiveness of QI methods’.

The 11 dimensions of leadership behaviour (Box 2), with responses ranging from 0 ‘never’ to 4 ‘very frequently’, were summed to form a scale. Total score for leadership behaviour ranged from 0 to 44 for each respondent. The scale for organizational culture for improvement comprised seven dimensions (Box 3), each ranging from 0 ‘very unsupportive’ to 10 ‘very supportive’, with a maximum score of 70. Twenty‐two QI methods with responses to use (‘have you used these QI methods, tools and techniques?’) and effectiveness (‘how often have these QI methods, tools and techniques led to changes in your service?’) ranging from 0 ‘not sure/never’ through to 3 ‘many times' (Box 4) were summed as ‘use of QI methods’ and ‘effectiveness of QI methods’ scales. Each had scores ranging from 0 to 66. In each case, raw scores were converted to percentages, giving a percentage score from 0% to 100%.

We used multiple regressions to examine the relationship between demographics, leadership behaviour and culture of innovation. We estimated the sample size assuming 1% (two‐tailed) significance level and 90% power to detect a small effect size (*f* ^2^ = 0.02) with up to eight dependent variables would require at least 1500 questionnaire responses. Statistical significance was set at 1% and data were analysed using Stata 12 [26]. Qualitative data were analysed using template analysis supported by NVivo 8 [27] and focused on responders' reported leadership behaviour, organizational culture of innovation and use of QI methods.

### Ethical approval

The study was approved by the National Research Ethics Service Committee East Midlands – Nottingham 1 (REC reference: 10/H0403/83) and the University of Lincoln School of Health and Social Care Ethics Committee.

## Results

### Demographic characteristics

From the 22 117 questionnaires sent out, we received 2743 responses (12%) from paramedics in the 11 participating ambulance services. Of the respondents 86 (3%) were ASCQI members (Table 1). There was no significant association between ASCQI membership and other attributes such as length of service, job role or number of colleagues that participants usually interacted with, questions forming scales describing leadership behaviour (Cronbach's alpha 0.87), culture of innovation (alpha 0.88), use of QI methods (alpha 0.91) and QI effectiveness (alpha 0.93) were internally consistent and were therefore converted to a percentage ‘score’ to simplify interpretation.

There was a strong positive correlation (0.77) between the score for the use of QI methods and QI effectiveness, that is, participants who used QI methods perceived these to be effective. The strength of other correlations ranged from weakly positive for organizational culture of innovation and leadership behaviour (0.19), to moderately positive: QI method score and culture of innovation (0.30); culture of innovation and leadership behaviour (0.40); QI effectiveness score and culture of innovation (0.28); QI effectiveness score and leadership behaviour (0.32). All correlations were statistically highly significant (*P* < 0.01).

### Leadership for improvement

In a multivariate analysis, leadership behaviour was significantly associated with ASCQI membership, length of service, job role and the number of colleagues that responders worked with (Table 2). Only 3% of responders were ASCQI members, but ASCQI members were significantly more likely to exhibit leadership behaviour compared with non‐ASCQI members (57.9% vs. 52.5%, *P* < 0.001).

**Table 2:** Factors associated with reported leadership behaviour, organizational culture of innovation and uptake of QI methods using multiple regression

		Coefficient	95% CI		*P* value
Leadership behaviour					
	ASCQI membership	5.17	2.01	8.33	**0.001**
	Years of service	0.67	0.31	1.02	<**0.001**
	Job role	0.78	0.43	1.13	<**0.001**
	Number of colleagues	1.94	1.39	2.48	<**0.001**
Innovation culture					
	ASCQI member	3.61	−0.50	7.72	0.085
	Years of service	−0.77	−0.31	−1.23	**0.001**
	Job role	0.16	−0.61	0.29	0.48
	Number of colleagues	0.79	0.087	1.50	**0.028**
Uptake of QI methods					
	Length of service	0.012	0.0036	0.021	**0.001**
	Emergency Care Assistant (EMT1)	0.22	0.15	0.30	<**0.001**
	Qualified Technician (EMT2)	0.11	0.049	0.17	<**0.001**
	Student Paramedic	−0.084	−0.15	−0.017	**0.013**
	Paramedic	0.10	0.054	0.15	<**0.001**
	Emergency Care Practitioner	0.15	0.07	0.22	<**0.001**
	Clinical/Paramedic Team Leader	0.19	0.13	0.25	<**0.001**
	Operational Manager	−0.056	−0.14	0.027	0.18
	10+ colleagues	−0.085	−0.13	−0.037	**0.001**

Many services had implemented models of clinical leadership, and these were perceived to be associated with improvement:

I feel that the [service] has made significant improvements since the introduction of a more clinical focused leadership model with Advanced Paramedics and Senior Paramedics. (Male, paramedic, non‐ASCQI member)

Staff felt that greater clinical engagement was critical to encouraging QI. To that end, they perceived that interaction with clinical leaders, providing greater opportunities to discuss clinical care with them, was crucial to facilitating the clinical engagement necessary for QI:

Clinical staff in my Trust have frequently shown willingness to engage in quality improvement, research and audit. I believe that if the leadership and resources are put in place then there would be widespread engagement and improved patient care as a result. (Male, clinical/paramedic team leader, non‐ASCQI member)

I feel staff should be given more time and appropriate opportunities to feed back what they have learnt from experience and get that feedback collated to assist other staff to make correct decisions. (Male, paramedic, ASCQI member)

### Culture of innovation

Responders' perception of their organizational culture of innovation was not significantly different between ASCQI members and those who were not (48.8% vs. 45.1%, *P* = 0.085). However, it was significantly associated with responders' length of service and the number of colleagues they had worked with (Table 2). Staff who had longer experience were more likely to perceive their organization to have a positive culture of innovation.

Many responders felt that their organizations were slow to change:

This service is very slow to react to new ideas in service improvement. (Male, paramedic, non‐ASCQI member)

Related to this, there was a perception that organizations lacked a culture of innovation. This had the added consequence of staff believing that there was little clinician engagement in QI, which was perceived to impede improvement itself:

I believe that we do not yet have a culture towards clinician‐led service improvement which leads to poorly‐thought‐out negative changes. (Male, paramedic, non‐ASCQI member)

The X Ambulance Service does not have an organisational culture that is supportive, in any way, in enabling ambulance clinicians to engage – effectively or meaningfully – in service and quality improvement. (Male, paramedic, ASCQI member)

The general perception was that organizations were slow to change and consequently were not particularly innovative. Specifically, some responders felt that targets were prioritized above innovation:

Ideas and innovations are frequently ignored in place of target‐based initiatives with little evidence base and no reward has ever been offered. (Male, emergency care assistant (EMT1), non‐ASCQI member)

It is difficult to achieve the aspirations of quality improvement initiatives when managerial interests in achieving operational targets continually clash and win. (Male, clinical team mentor, ASCQI member)

Many responders cited factors that prevented greater clinical involvement in QI initiatives. A major barrier was a lack of time, resources or support for personal development, which meant front‐line staff often undertook training in their own time and at their own expense. Limited rewards and career progression negatively affected morale. Problems with equipment were also cited as a barrier to quality and QI.

To achieve and maintain clinician involvement, staff should not be expected to be involved in their own free time. Too much time and effort is given without acknowledgement or reward. (Female clinical/paramedic team leader, ASCQI member)

I cannot remember a shift recently when I have not found a significant piece of equipment unserviceable or out of date (at ambulance stations not air bases). This is because a sufficient amount of time at the start of the shift is not given to check drugs and equipment. (Male, critical care paramedic, non‐ASCQI member)

Although many respondents reported a lack of evidence for a culture of innovation a minority disagreed with this.

Working with X Ambulance Service I have been positively supported with developing product innovation. (Male, emergency care practitioner, non‐ASCQI member)

Some staff made recommendations about how to drive improvement. Here, one responder emphasized a need for greater patient feedback:

All patients who receive the care bundles should be identified and followed up and their outcome delivered to the staff who gave their care; ambulance crews have great difficulty in finding out any outcome of patient journey and results of interventions as there are patient confidentiality and data access barriers. If staff could be given a short summary of the patient outcome from the receiving hospital/GP – maybe they would then relate it to an actual patient they treated and not just see it as a paper/tick box exercise they are told they should do to obtain a ‘target’. (Female, emergency care practitioner, non‐ASCQI member)

### Uptake of QI methods

Uptake of QI methods was generally low, but significantly higher among ASCQI than non‐ASCQI members (15.8% vs. 9.1%, *P* < 0.001). Uptake of QI methods was significantly associated with length of service. Paramedics, team leaders and operational managers were most likely to use QI methods in contrast to student paramedics or those with more than 10 colleagues, who were least likely to use QI methods (Table 2).

Low uptake of QI methods was due to conflicting priorities and operational pressures.

In a time of rota changes and pension uncertainties, in my place of work at least, trying to get any clinician to engage in any improvement initiatives with any degree of enthusiasm is nigh on impossible. There are of course, the interested few, but these are a minority and are largely unsupported. (Female, paramedic, non‐ASCQI member)

Although most qualitative responses reinforced a picture of low uptake of QI methods, there were exceptions.

Clinical staff in my Trust have frequently shown willingness to engage in quality improvement, research and audit. (Male, clinical/paramedic team leader, non‐ASCQI member)

## Discussion

### Summary of results

Leadership behaviour was significantly higher for ASCQI members (i.e. those directly involved in the QIC) than for non‐ASCQI members. This could have been due to the ASCQI attracting clinicians who expressed leadership behaviours, or involvement in the ASCQI encouraging this behaviour or both.

ASCQI members were also significantly more likely to use QI methods and the use of QI methods was also significantly associated with leadership behaviour. Direct involvement in the ASCQI did not significantly affect responders' perceptions of the culture of innovation of their organization, which was generally considered to be poor.

The specific objectives to improve the delivery of AMI and stroke care bundles in England were achieved by the end of the project and improvements were likely to be due to the QIC emphasizing clear goals, individualized or team feedback, and system changes in successful services such as provider prompts, engagement with front‐line clinicians and shared learning between participants and organizations [21].

Another purpose of the QIC was to attract clinicians to engage in the work of the collaborative and to spread learning about QI methods through formal and informal peer‐to‐peer networks within and between services. While only 3% of respondents were ASCQI members, the QIC did introduce QI methods into ambulance services through workshops, seminars, and written information delivered by small teams of clinical audit staff and QI champions [21].

While front‐line staff perceived a disconnect between their priorities (e.g. better patient care) and those of managers (e.g. meeting targets), there were constructive suggestions, about how to promote leadership, for example, through greater managerial commitment to personal development, as well as to implement and embed QI into ambulance service practices, for example, giving front‐line clinicians greater opportunities to effect the necessary changes.

Therefore, we hypothesize that QI methods led to improvement in individual ambulance services through an improvement subculture, which was mediated by the ASCQI. The improvement subculture comprised local clinical leaders engaging teams of staff in learning QI techniques and applying these to overcome barriers to the delivery of care for AMI and stroke. The improvements were achieved despite a perception among staff of a poor organizational culture of innovation and low uptake of QI methods more generally.

### Strengths and weaknesses

This study was a national survey involving 11 of the 12 English ambulance services. Strengths included the large number of responses, which enabled us to examine relationships between key variables, explore free‐text responses using qualitative methods and thus, triangulate with the quantitative data. The response rate (12%) was low, reflecting problems for paramedics in accessing the online survey during or between shifts, and a lack of time or interest. Responders may have been more positive or more negative in their views. Although this could affect generalizability, the proportion of services included and triangulation with analysis of free‐text responses supports our conclusions.

### Comparison with existing literature

This study confirms that leadership and knowledge of QI methods were associated with involvement in a successful QIC [15]. Our findings contrast with previously accepted notions that organizational culture of innovation is a prerequisite for improvement by showing that performance improvement can occur despite a background of poor organizational culture of innovation if groups or subgroups within the organization are sufficiently empowered to improve quality.

The contribution of context to the likelihood of success of QI efforts is becoming better understood. Context includes the wider environment and organisational characteristics (the macro‐system); the individual provider and their role in the organization (the clinical micro‐system); as well as the external QI team providing support to a QI project [7].

Clinical leadership and organizational culture are important features of this context through the organizational macro‐system, the clinical micro‐system and the QI support provided. Ambulance services are macro‐systems, which have been characterized in the past as command‐and‐control, risk‐averse organizations [28]. The command‐and‐control culture may have led previously to a management style reflected in the negative staff perceptions in our study of the innovation culture of their organizations. This negative perception was inconsistent with strong executive and management support for the ASCQI.

Recent reforms have sought to professionalize ambulance services through Health Professions Council status for paramedics; paramedic degrees; advanced practitioner and consultant paramedic status; the opening up of non‐medical clinical academic careers [2]; and the general move from a managerial to a clinical professional culture [14]. In contrast, there were clear clashes in this study between the ‘executive’ (management) and ‘operator’ (front‐line clinician) subcultures within the organizations [29, 30]. This was expressed through staff perceptions of organizations not being clinically‐led, lack of support for learning, limited rewards for staff and a ‘target‐driven culture’, that prioritizes response time targets ahead of patient care [28].

Despite respondents in this study perceiving an organization that was sometimes unsupportive and lacking a culture of innovation, significant innovation did occur in the ASCQI, as evidenced by significant improvements in measured care for AMI and stroke. This may have been due to a variety of factors including QI expert input; effective clinical leadership; care bundles being seen as useful and relevant; and the critical mass of QI teams working on the project. We hypothesize that these factors, together with inputs such as effective interaction, communication and feedback [31], enabled a sufficient improvement subculture to develop, which helped facilitate widespread adoption of AMI and stroke care bundles for [17, 20]. The extent to which information was communicated was a key issue. Effective communication channels fostered a shared ethos, which increased the likelihood of innovation and change [30].

The ASCQI used a participative style of leadership with front‐line clinicians being given greater control and empowered to change processes [32]. They were given the tools to improve care processes and given the autonomy to test new ideas. While data were being used to evaluate the interventions implemented, those facilitating the QIC were keen for the project not to be ‘target‐driven’. Clinicians were motivated through a focus on benefitting patient through the use of care bundles, rather than achieving targets.

Evidence has shown that the stronger the desirability of a certain outcome, and the more people believe that their efforts are instrumental in achieving that outcome, the stronger the person will be motivated to do what is required. This may also explain the improvements achieved through the ASCQI [33].

### Implications for practice, policy and future research

An organizational culture of innovation and widespread knowledge of QI methods are often considered crucial to achieving QI, but appeared to be lacking in this study. Perhaps surprisingly, this did not seem to impede this QIC. Instead, we found evidence of an improvement subculture sufficiently able to mediate large‐scale change through leadership and use of QI methods.

Despite this improvement, for QI to be sustained and continued within health care organizations, it will be important to unite the seemingly divergent priorities of management and clinical staff. This necessitates better communication between the two; QI has to involve clinician and management input into and engagement with QI.

A culture of innovation and QI also requires greater management commitment to, and investment in, training and equipment to support improvement efforts. This may, in turn, address staff turnover and career progression issues. Previous research on QI comes from the practitioner perspective. Given that patients are key intended beneficiaries, a better understanding of their experiences and needs should inform QI. We still do not fully understand success factors for QI and this requires further research [20].

## Conclusions

An organizational culture of innovation, often considered a prerequisite for successful QI, was lacking in many ambulance services. Despite this, and the low uptake of QI methods, the QIC achieved its objective to improve pre‐hospital care for heart attack and stroke. Further research needs to done to understand success factors for QI in different health care contexts.

## Conflict of interest

The authors declare no conflict of interest.

## Author contributions

ANS had the original idea for the study. ANS, NE and AS were involved in the design of the study. NE collected the study data. VHP, ZA and AS were involved in data analysis. All authors were involved in the interpretation and discussion of results. All authors contributed to the writing and review of the various drafts of the report. ANS is the guarantor of the study.

## Supporting information

Supporting information
