# Supplementary material for: Exploration of contextual factors in a successful quality improvement collaborative in English ambulance services: cross‐sectional survey
Source: J Eval Clin Pract. 2015 Aug 24;22(1):77–85. doi: 10.1111/jep.12438 (PMC5215672; doi:10.1111/jep.12438)
Supplement: Supplementary file 1 — Supporting information [file JEP-22-77-s001.pdf]

Appendix S1

Ambulance service cardiovascular quality initiative: QI survey

This questionnaire is part of the Ambulance Service Cardiovascular Quality Initiative (ASCQI) project; a large national project which aims to involve A&E operational frontline ambulance clinicians in quality improvement in cardiovascular pre-hospital care.

The objective of this questionnaire is to obtain an understanding of current quality improvement behaviour, culture and methods used in the twelve ambulance services in England. The information received will be used to influence and support clinician engagement in the future development and delivery of pre-hospital care.

The ASCQI project team would be most grateful if you would spare some time to complete this questionnaire. Most questions only require you to tick a box and should take no more than 15 minutes to complete.

Please note that participation is voluntary. Your responses are anonymous and all the answers you provide will be treated as completely confidential.

NB: The survey should be completed by A&E Operational frontline clinicians only

Background Information

Q1 **What is your gender?**

Male ☐ Female ☐

Q2 **How many years have you been an operational A&E clinician in the ambulance service?**

0–4 ☐ 5–9 ☐ 10–14 ☐ 15–19 ☐ 20–24 ☐ 25+ ☐

Q3 **What is your current role in the ambulance service?**

Emergency Care Assistant (EMT1) ☐
Qualified Technician (EMT2) ☐
Student Paramedic ☐
Paramedic ☐
Emergency Care Practitioner ☐
Clinical/Paramedic Team Leader ☐
Operational Manager ☐

Q4 **Where are you based? *(Please select the location which describes where you work the majority of the time)***

Ambulance station (at main group station) ☐
Ambulance station (at sub-station) ☐
Ambulance station (at Trust/regional HQ) ☐
Ambulance station (at divisional/sector HQ) ☐
Office based (at Trust/regional HQ) ☐
Office based (at divisional/sector HQ) ☐
Office based (at main group station) ☐
Office based (as sub-station) ☐

Q5 **How many colleagues do you usually interact with on daily basis?**

0–3 ☐ 4–6 ☐ 7–9 ☐ 10+ ☐

Q6 **Are you a member of the Ambulance Services Cardiovascular Quality Initiative (ASCQI) collaborative?**

Yes ☐ No ☐

Q7 **Which ambulance service do you work for?**

East Midlands ☐
East of England ☐
Great Western ☐
Isle of Wight ☐
London ☐
North East ☐
North West ☐
South Central ☐
South East Coast ☐
South Western ☐
West Midlands ☐
Yorkshire ☐

Quality Improvement Behaviour

Questions 8–18 relate to quality improvement behaviour.

Please read each question carefully and ask yourself how frequently you engage in the behaviour described and select the appropriate box.

Please be realistic about the extent to which you actually engage in the behaviour and answer in how you typically behave on most days, on most tasks and with most people.

If you feel the question doesn't apply to you, it is probably because you do not frequently engage in the behaviour described. In that case assign a rating of 3 or less.

Q8 **How often do you talk to others about future trends that will influence how your work gets done?**
Never ☐ Rarely ☐ Sometimes ☐ Frequently ☐ Very Frequently ☐

Q9 **How often do you seek out new challenges or opportunities that test your skills and abilities?**
Never ☐ Rarely ☐ Sometimes ☐ Frequently ☐ Very Frequently ☐

Q10 **How often do you describe an image to others of what your future ambulance service could be like?**
Never ☐ Rarely ☐ Sometimes ☐ Frequently ☐ Very Frequently ☐

Q11 **How often do you challenge others to try out new and innovative ways to do their work?**
Never ☐ Rarely ☐ Sometimes☐ Frequently ☐ Very Frequently ☐

Q12 **How often do you ask others to share their aspirations of their future within the ambulance service?**
Never ☐ Rarely ☐ Sometimes ☐ Frequently ☐ Very Frequently ☐

Q13 **How often do you search outside of your own organization for ways to improve what you do?**
(e.g. look at other organizations or people to see how they work)
Never ☐ Rarely ☐ Sometimes ☐ Frequently ☐ Very Frequently ☐

Q14 **How often do you show others how long-term interests at work can be realized by sharing a common vision?**
(e.g. do you have a clear vision of the future and do you communicate this to others?)
Never ☐ Rarely ☐ Sometimes ☐ Frequently ☐ Very Frequently ☐

Q15 **How often do you ask 'what can I learn?' when things don't go as expected?**
Never ☐ Rarely ☐ Sometimes ☐ Frequently ☐ Very Frequently ☐

Q16 **How often do you share with others what you want to achieve in your role?**
Never ☐ Rarely ☐ Sometimes ☐ Frequently ☐ Very Frequently ☐

Q17 **How often do you make certain you set achievable goals, make accurate plans and establish measurable milestones for the tasks you work on?**
Never ☐ Rarely ☐ Sometimes ☐ Frequently ☐ Very Frequently ☐

Q18 **How often do you speak with your colleagues about the meaning and purpose of your work?**
Never ☐ Rarely ☐ Sometimes ☐ Frequently ☐ Very Frequently ☐

Organizational Culture for Innovation Assessment Rating Scale

Questions 19–25 relate to organizational culture and leadership that support innovation. We would like to know what degree of skills, systems and organizational experience there is available to you to support innovation.

Please read each question carefully before selecting your answer.

Q19 **RISK TAKING:** To what degree does your organization provide support for you to try out something new (given that reasonable precautions to avoid harm to patients or disruptions to the organization have been made)?
+5 VERY SUPPORTIVE ☐ +4 ☐ +3 ☐ +2 ☐ +1 ☐ 0 NEITHER SUPPORTIVESUPPORTIVE OR UNSUPPORTIVE ☐ –1 ☐ –2 ☐ –3 ☐ –4 ☐ –5 VERY UNSUPPORTIVE ☐

Q20 **RESOURCES FOR INNOVATION:** To what degree does your organization provide money, protected time, information and/or authority to act for those who wish, to try new ways of working?
+5 VERY SUPPORTIVE ☐ +4 ☐ +3 ☐ +2 ☐ +1 ☐ 0 NEITHER SUPPORTIVESUPPORTIVE OR UNSUPPORTIVE ☐ –1 ☐ –2 ☐ –3 ☐ –4 ☐ –5 VERY UNSUPPORTIVE ☐

Q21 **WIDELY SHARED KNOWLEDGE:** To what degree is knowledge gathered and easily shared throughout your organization?
+5 VERY SUPPORTIVE ☐ +4 ☐ +3 ☐ +2 ☐ +1 ☐ 0 NEITHER SUPPORTIVESUPPORTIVE OR UNSUPPORTIVE ☐ –1 ☐ –2 ☐ –3 ☐ –4 ☐ –5 VERY UNSUPPORTIVE ☐

Q22 **SPECIFIC TARGETS:** To what degree do your managers make it clear that new and better ways of working are important in areas that are strategically or operationally important to the organization?

+5 VERY SUPPORTIVE ☐ +4 ☐ +3 ☐ +2 ☐ +1 ☐ 0 NEITHER SUPPORTIVESUPPORTIVE OR UNSUPPORTIVE ☐ –1 ☐ –2 ☐ –3 ☐ –4 ☐ –5 VERY UNSUPPORTIVE ☐

Q23 **TOOLS & TECHNIQUES:** To what degree does your organization actively support and promote the use of quality improvement methods?

+5 VERY SUPPORTIVE ☐ +4 ☐ +3 ☐ +2 ☐ +1 ☐ 0 NEITHER SUPPORTIVESUPPORTIVE OR UNSUPPORTIVE ☐ –1 ☐ –2 ☐ –3 ☐ –4 ☐ –5 VERY UNSUPPORTIVE ☐

Q24 **REWARD SYSTEMS:** To what degree does your organization reward the innovative efforts of individuals by giving these people things that they really want? (e.g. more protected time for research, more authority and recognition amongst peers etc)

+5 VERY SUPPORTIVE ☐ +4 ☐ +3 ☐ +2 ☐ +1 ☐ 0 NEITHER SUPPORTIVESUPPORTIVE OR UNSUPPORTIVE ☐ –1 ☐ –2 ☐ –3 ☐ –4 ☐ –5 VERY UNSUPPORTIVE ☐

Q25 **RAPIDLY FORMED RELATIONSHIPS:** To what degree does your organization easily form high-performing teams and networks of motivated individuals?

+5 VERY SUPPORTIVE ☐ +4 ☐ +3 ☐ +2 ☐ +1 ☐ 0 NEITHER SUPPORTIVESUPPORTIVE OR UNSUPPORTIVE ☐ –1 ☐ –2 ☐ –3 ☐ –4 ☐ –5 VERY UNSUPPORTIVE ☐

Quality Improvement Methods, Tools and Techniques

Have you ever used any of these quality improvement methods, tools and techniques?		Not sure	Never	Infrequently	Sometimes	Many times	*If so, how often has this led to changes in your service?*	Not sure	Never	Infrequently	Sometimes	Many times
Q27	Clinical audit	☐	☐	☐	☐	☐	Q27a	☐	☐	☐	☐	☐
Q28	PDSA cycles (Plan-do-study-act)	☐	☐	☐	☐	☐	Q28a	☐	☐	☐	☐	☐
Q29	Significant event analysis	☐	☐	☐	☐	☐	Q29a	☐	☐	☐	☐	☐
Q30	Root cause analysis	☐	☐	☐	☐	☐	Q30a	☐	☐	☐	☐	☐
Q31	SWOT/SCOT analysis (strength, weaknesses, challenges, opportunities and threats)	☐	☐	☐	☐	☐	Q31a	☐	☐	☐	☐	☐
Q32	Force field analysis	☐	☐	☐	☐	☐	Q32a	☐	☐	☐	☐	☐
Q33	Process mapping	☐	☐	☐	☐	☐	Q33a	☐	☐	☐	☐	☐
Q34	Process redesign	☐	☐	☐	☐	☐	Q34a	☐	☐	☐	☐	☐
Q35	WIFM charts ('whats in it for me?')	☐	☐	☐	☐	☐	Q35a	☐	☐	☐	☐	☐
Q36 Financial rewards for staff		☐	☐	☐	☐	☐	Q36a	☐	☐	☐	☐	☐
Q37	Role redesign	☐	☐	☐	☐	☐	Q37a	☐	☐	☐	☐	☐
Q38	Confidence charts (funnel plots)	☐	☐	☐	☐	☐	Q38a	☐	☐	☐	☐	☐
Q39	Run/control charts	☐	☐	☐	☐	☐	Q39a	☐	☐	☐	☐	☐
Q40	Pareto charts	☐	☐	☐	☐	☐	Q40a	☐	☐	☐	☐	☐

Q41	Cause and effect diagrams	☐	☐	☐	☐	☐	Q41a	☐	☐	☐	☐	☐
Q42	Swim lane diagrams	☐	☐	☐	☐	☐	Q42a	☐	☐	☐	☐	☐
Q43	CTQ trees (critical-to-quality)	☐	☐	☐	☐	☐	Q43a	☐	☐	☐	☐	☐
Q44	Patient interviews	☐	☐	☐	☐	☐	Q44a	☐	☐	☐	☐	☐
Q45	Focus groups	☐	☐	☐	☐	☐	Q45a	☐	☐	☐	☐	☐
Q46	Balanced scorecards	☐	☐	☐	☐	☐	Q46a	☐	☐	☐	☐	☐
Q47	Lean	☐	☐	☐	☐	☐	Q47a	☐	☐	☐	☐	☐
Q48	Six sigma	☐	☐	☐	☐	☐	Q48a	☐	☐	☐	☐	☐

Q26 Lastly, we welcome any additional comments or suggestions you may have of how to achieve and maintain clinician engagement in quality improvement initiatives.

Suggestions:

Thank you for taking the time to complete this questionnaire.
